# Clinical Outcomes Depending on Sympathetic Innervation in Pancreatic Cancer

**DOI:** 10.3390/cancers15113040

**Published:** 2023-06-02

**Authors:** Elena-Anca Târtea, Mihai Petrescu, Ion Udriștoiu, Victor Gheorman, Viorel Biciușcă, Alexandra-Roxana Petrescu, Ana-Maria Ciurea, Cristin Constantin Vere

**Affiliations:** 1 Department of Neurology, University of Medicine and Pharmacy of Craiova, 200349 Craiova, Romania; anca.tartea@umfcv.ro; 2 Department of Psychiatry, University of Medicine and Pharmacy of Craiova, 200349 Craiova, Romania; ion.udristoiu@umfcv.ro (I.U.); victor.gheorman@umfcv.ro (V.G.); 3 Department of Internal Medicine, University of Medicine and Pharmacy of Craiova, 200349 Craiova, Romania; biciuscaviorel@gmail.com; 4 Department of Pathology, University of Medicine and Pharmacy of Craiova, 200349 Craiova, Romania; alexandra.ciuciulete@yahoo.com; 5 Department of Oncology, University of Medicine and Pharmacy of Craiova, 200349 Craiova, Romania; amciurea14@gmail.com; 6 Department of Gastroenterology, University of Medicine and Pharmacy of Craiova, 200349 Craiova, Romania; cristin.vere@umfcv.ro

**Keywords:** pancreatic cancer, sympathetic nerve fibers, beta 2 adrenoreceptors, clinical outcomes

## Abstract

**Simple Summary:**

Pancreatic cancer, one of the most aggressive forms of cancer, is a lethal disease. Although nerves infiltrate the tumor microenvironment and they are even present in pancreatic tumors, the role of innervation, especially sympathetic innervation, remains unclear. Moreover, neurotransmitters can modulate the evolution of pancreatic cancer through various processes, such as carcinogenesis, invasions, proliferation, or metastasis. Here, we assess pancreatic neoplastic tissue, examining the sympathetic nerve fibers and the density of beta 2 adrenoreceptors, both intratumoral and peritumoral. These were correlated with the clinical outcome. If the intratumoral sympathetic nerves registered a structural alteration, and the peritumoral nerves were not structurally altered, they could not be associated with prognosis; the same was not observed regarding beta 2 adrenoreceptors. Increased immunoreactivity of beta 2 adrenoreceptors in pancreatic peritumoral tissue was associated with poor prognostic factors in pancreatic cancer.

**Abstract:**

Background: The aim of our study was to evaluate sympathetic neuronal remodeling in patients with pancreatic cancer, together with its correlation with clinical outcomes. Methods: In this descriptive, retrospective study, we analyzed pancreatic cancer specimens and peritumoral pancreatic tissue from 122 patients. We also investigated tyrosine hydroxylase immunoreactivity for the analysis of sympathetic nerve fibers and beta 2 adrenoreceptors immunoreactivity. To investigate the potential interaction between tyrosine hydroxylase (TH), beta 2 adrenoreceptors (B2A) immunoreactivity, and clinicopathological outcomes, we used the median to classify each case as TH+, respectively, B2A+ (if it presented a value higher than the median). Results: Firstly, the overall survival was analyzed according to TH and B2A immunoreactivity, in both intratumoral and peritumoral tissue. Only B2A immunoreactivity in the peritumoral pancreatic tissue influenced overall survival at 5 years of follow-up; thus, B2A+ patients recorded a 5-year survival of only 3% compared to B2A− patients who recorded an overall survival at 5 years of follow-up of 14% (HR = 1.758, 95% CI of ratio 1.297 to 2.938, *p* = 0.0004). Additionally, the increased immunoreactivity of B2A in the peritumoral tissue was also associated with other factors of poor prognosis, such as moderately or poorly differentiated tumors, the absence of response to first-line chemotherapy, or metastatic disease. Conclusions: The increased immunoreactivity of beta 2 adrenoreceptors in pancreatic peritumoral tissue represents a poor prognostic factor in pancreatic cancer.

## 1. Introduction

Pancreatic cancer is one of the most aggressive forms of cancer, with a 5-year survival rate of approximately 12.5% according to the latest data published in 2023 by the National Cancer Institute from the United States of America [1]. According to the latest data from the International Agency for Research on Cancer (World Health Organization), the global incidence of pancreatic cancer in 2020 was 4.9 per 100,000 for both sexes, for all age groups, with a mortality rate of almost 4.5 per 100,000 for both sexes, all age groups [2]. Due to the poor survival rates, the population distribution of people who die of pancreatic cancer is similar to that of people who are diagnosed with the disease. It is estimated that there will be 64,050 new cases of pancreatic cancer in the USA in 2023, and approximately 50,550 people will die of this disease [1].

Although nerves infiltrate the tumor microenvironment of pancreatic cancer and contribute to tumor progression, the pathological role of tumor innervation remains unclear [3].

In particular, the sympathetic innervation of pancreatic tumors has emphasized the role of the sympathetic nervous system in the pathophysiology and clinical evolution (for example severity of pain) of this disease [4]. Renz B. et al. recently conducted an animal model study which demonstrated that stimulating beta 2 adrenoreceptors by increasing circulating systemic epinephrine levels promoted kras-induced pancreatic tumorigenesis. Moreover, catecholamines promoted PDAC development, nerve growth factor (NGF) secretion, and pancreatic nerve density [5].

The pancreas contains both extrinsic and intrinsic innervation through sympathetic and parasympathetic fibers. Nerve fibers exit or enter the pancreas, then disseminate in the stromal septa and comprise the pancreatic stroma [6]. Firstly, by means of nerve fibers, neoplastic cells can metastasize towards lymphatic vascular invasion [7]. Neural invasion has a high incidence in pancreatic cancer among gastrointestinal malignancies, but there are few criteria that quantify this parameter, and the information is often vague [8]. On the other hand, the first to hypothesize a paracrine molecular signaling mechanism between cancerous pancreatic cells and nerves, explaining pancreatic cancer nerve affinity, was Bockman et al. [9]. In this study, it is specified that pancreatic adenocarcinoma is not limited to the periphery of the nerves, but penetrates the perineurium and becomes intimately associated with Schwann cells and axons in the endoneurium [9]. Thus, a deterioration of the neural elements occurs. Moreover, the transforming growth factor alpha (TGF-alpha) is abundant in pancreatic nerves, and the epidermal growth factor receptor (EGFR) is prominent in adenocarcinoma cells, constituting a growth advantage of pancreatic adenocarcinoma [9]. Moreover, neurotransmitters can modulate the development of pancreatic cancer in multiple aspects, such as carcinogenesis, invasions, proliferation, or metastasis [10,11,12]. A feedforward signaling loop was recently reported in the mouse model of PDAC, whereby beta 2 adrenoreceptors signaling leads to upregulation of NGF secretion, fueling increased axogenesis and tumor growth [5]. However, before this study, it was reported in the premalignant stage of a genetically engineered murine PDAC model, demonstrating the occurrence of an early axonogenic influence on carcinogenesis [13]. Moreover, the genomic evaluation of human PDAC highlighted somatic aberrations in embryonic regulators of axon guidance, such as SLIT/ROBO signaling [14], highlighting a role for neuronal modulation in pancreatic carcinogenesis. However, there are few studies that analyze the distribution of nerve fibers in pancreatic cancer, especially the sympathetic nerve fibers and beta 2 adrenoreceptors [3,5,15]. Moreover, understanding the molecular mechanisms of the interaction between nerves and pancreatic cancer could represent an attractive therapeutic target.

The aim of our study was to assess sympathetic neuronal fibers and beta 2 adrenoreceptors in patients with pancreatic cancer and their correlation with clinical outcomes.

## 2. Materials and Methods

### 2.1. The Type of Study

We performed a descriptive, retrospective study which included 122 patients diagnosed and treated for pancreatic cancer between 2014 and 2017. All patients received a follow-up of 5 years or until their death. This study was approved by the Ethics Committee of the University of Medicine and Pharmacy of Craiova (No. 225/20.12.2021), and conducted in accordance with the Helsinki Declaration and other international regulations. Each patient included in our study signed their consent, meaning that data from their medical records can be used for research purposes. No data could be used to identify a patient because each patient received a specific code and all identifying information was deleted from the study database.

### 2.2. Patients and Tissues

In our study, fragments of pancreatic neoplastic tissue taken after the resection of a pancreatic tumor were analyzed. All patients were informed that the tissue fragments taken would be used to study nerve influences in this type of cancer. Initially, pancreatic ductal adenocarcinoma (PDAC) was the major subtype of this cancer; minor subtypes included ductal adenocarcinoma/partially mucinous adenocarcinoma, ductal adenocarcinoma/adenosquamous carcinoma, or adenosquamous carcinoma. However, ultimately, for the homogeneity of the results, the samples were completely analyzed only from patients with PDAC. The available clinicopathological features were as follows: patient gender and age, survival status, histological subtypes, tumor grading, smoking history, lymphatic metastasis status, and location.

### 2.3. Immunohistochemistry

Pancreatic tissue samples were collected from patients after pancreatic resection. The resected pancreatic tissue samples were divided into sections that were immediately fixed in 4% paraformaldehyde, followed by paraffin embedding. Consecutive 4 μm-thick sections from paraffin-embedded samples were analyzed to identify sympathetic influences. Intra- and peritumoral pancreatic sympathetic nerve fibers were analyzed using Tyrosine Hydroxylase Antibody (TH, dilution 1:20; Novus Biological, Abingdon, UK), and in tumor and peritumoral tissue, the immunoreactivity of beta 2 adrenergic receptors was also analyzed (dilution 1:100; Novus Biological, Abingdon, UK) using a primary antibody. The slides were further incubated with monoclonal primary antibody at 4 °C for 18 h. Finally, the signal was located via 3,3′-diaminobenzidines (DAB) (Dako, Glostrup, Denmark). After hematoxylin and eosin staining, the slides were cover-slipped in DPX (Sigma-Aldrich, St. Louis, MO, USA).

### 2.4. Quantitative Analysis of Neural Tissue

Sympathetic nerve fibers were analyzed using tyrosine hydroxylase cross-sectional area (% of the area of a nerve), and for beta 2 adrenergic receptors, we used integrated optical density (IOD). Images were acquired using a Nikon Eclipse 90i microscope (Elta90, Bucharest, Romania) equipped with a Nuance FX multispectral camera and Nuance analysis software (Perkin Elmer, Hopkinton, MA, USA). Multispectral microscopy was used to analyze the percentage area of tyrosine hydroxylase in the nervous tissue area, as well as to calculate the integrated optical density (IOD) of the beta 2 adrenergic receptors in the tumor tissue and in the peritumoral tissue. After collecting spectral images from individual sections stained with DAB or hematoxylin–eosin, we performed unmixing efficiency of the color channels and characterized the immunohistochemical expression of the pattern of interest. The corresponding color channel was then analyzed with Image ProPlus AMS 7 software (Media Cybernetics, Bethesda, MD, USA). To assess the sympathetic fibers, the area of TH in each nerve was calculated, both in the tumor and in the peritumoral tissue. Later, the mean and standard deviation of a mean of TH in the tumor, respectively peritumoral, was shown. Regarding beta 2 adrenoreceptors, 5 random images were analyzed in the tumor tissue and another 5 random images from the peritumoral pancreatic tissue. The analysis was carried out independently by 2 pathologists with experience in this field. Similar to TH, for beta 2 adrenoreceptors, the mean and the standard deviation of the mean of IOD were shown; both resulted from the analysis of the 5 random images from the tumor, respectively peritumoral. The median of tyrosine hydroxylase immunoreactivity per nerve, respectively, of beta 2 adrenergic receptor immunoreactivity was used to analyze the survival of patients with pancreatic cancer.

### 2.5. Statistical Analysis

All statistical analysis was performed using GraphPad software (Version 9.5, La Jolla, CA, USA). To evaluate the statistical differences between two groups, we used Student’s *t*-test and the analysis of variance ANOVA (analysis of variance). Results were expressed as mean ± standard deviation of the mean (s.d.m.). Using Kaplan–Meier curves, we analyzed patient survival according to each variable, using the log-rank test to establish statistical significance. Correlation between TH or B2A and other pathological variables were performed using Fisher’s exact test. In all cases, *p* < 0.05 was considered statistically significant.

## 3. Results

### 3.1. Immunoreactivity of Tyrosine Hydroxylase and Beta 2 Adrenergic Receptors in Pancreatic Cancer and Peritumoral Tissues

**Figure 1:**
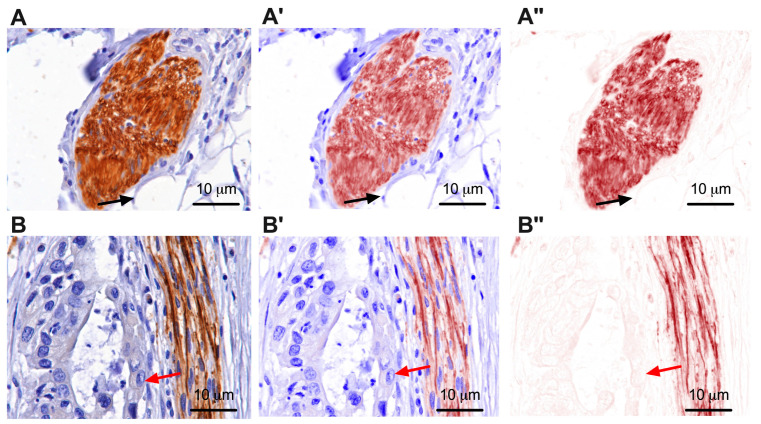
Representative pictures with sympathetic fibers in pancreatic peritumoral nerves (**A**), respectively, with sympathetic fibers in pancreatic intratumoral nerves (**B**), immunoreactivity for tyrosine hydroxylase, 40×. (**A’**,**B’**) represent the mixed spectral compound. (**A’’**,**B”**) represent the color channel only for tyrosine hydroxylase. Black arrows indicate adipose tissue around the nerve. Red arrows indicate pancreatic neoplastic cells around the nerve. Representative pictures with sympathetic fibers in pancreatic peritumoral nerves, respectively, with sympathetic fibers in pancreatic intratumoral nerves at a smaller scale (4× and 10×) are shown in Supplementary Figures S1–S4.

**Figure 2:**
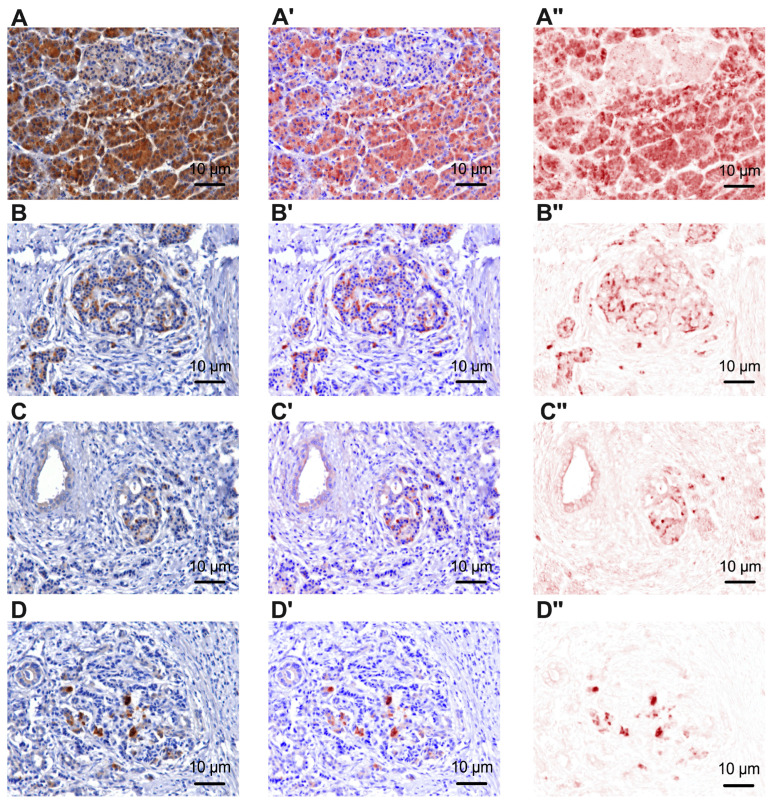
Representative pictures with the immunoreactivity of beta 2 adrenergic receptors in peritumoral pancreatic tissue (**A**) and in well-differentiated—G1 (**B**), moderately differentiated—G2 (**C**) and poorly differentiated—G3 (**D**) pancreatic tumor tissue, 40×. (**A’**–**D’**) represent the mixed spectral compound. (**A’’**–**D”**) represent the color channel only for beta 2 adrenergic receptors. In the additional figures (Figures S5 and S6), representative pictures with low magnificence (4× and 10×) are shown where tumor area and peritumor area are indicated.

This study analyzed pancreatic cancer specimens and peritumoral pancreatic tissue from 122 patients. The immunoreactivity of tyrosine hydroxylase for the analysis of sympathetic nerve fibers (Figure 1 and Supplementary Figures S1–S4) and the immunoreactivity of beta 2 adrenergic receptors (Figure 2) were investigated.

**Figure 3:**
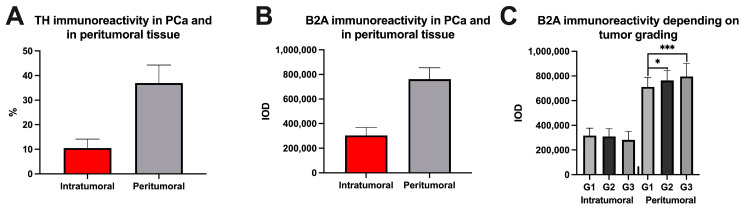
Immunoreactivity in pancreatic cancer (PCa) and in peritumoral tissue for tyrosine hydroxylase (TH)—(**A**), beta 2 adrenoreceptors (B2A)—(**B**) and for B2A depending on tumor grading—(**C**). In Figure 3A the analysis included 348 nerves from tumor tissues and 865 nerves from peritumoral tissues. In Figure 3B, the intratumoral group included the analysis of 594 samples and the peritumoral group included the analysis of 471 samples. In Figure 3C, the intratumoral group included the analysis of 123 samples for G1, 298 for G2, and 173 samples for G3, while the peritumoral group included the analysis of 97 samples for G1, 236 for G2, and 138 for G3. * represents statistical significance and *** represents extremely significant.

We observed a reduction in tyrosine hydroxylase immunoreactivity in neoplastic tissue to 10.48 ± 3.66% compared to tyrosine hydroxylase immunoreactivity in peritumoral tissue, where a value of 36.93 ± 7.32% was recorded (*p* < 0.0001, Figure 3A). In addition to the analysis of tyrosine hydroxylase immunoreactivity, the immunoreactivity of beta 2 adrenergic receptors was analyzed in pancreatic peritumoral tissue and tumor tissue. A higher value of these receptors was recorded in the peritumoral tissue (303336±65383 intratumorally vs. 761,648 ± 93,188, *p* < 0.0001, Figure 3B). In this analysis, significant differences were observed in the immunoreactivity of peritumoral beta 2 adrenergic receptors, depending on the tumor grading as follows: G1 vs. G2 (710,854 ± 78,174 vs. 763,575 ± 81,950, *p* = 0.0338) and G1 vs. G3 (710,854 ± 78,174 vs. 796,024 ± 106,601, *p* = 0.0010) (Figure 3C). It should be mentioned that between G2 vs. G3, no significant statistical difference was recorded (763,575 ± 81,950 vs. 796,024 ± 106,601, *p* = 0.2020).

**Figure 4:**
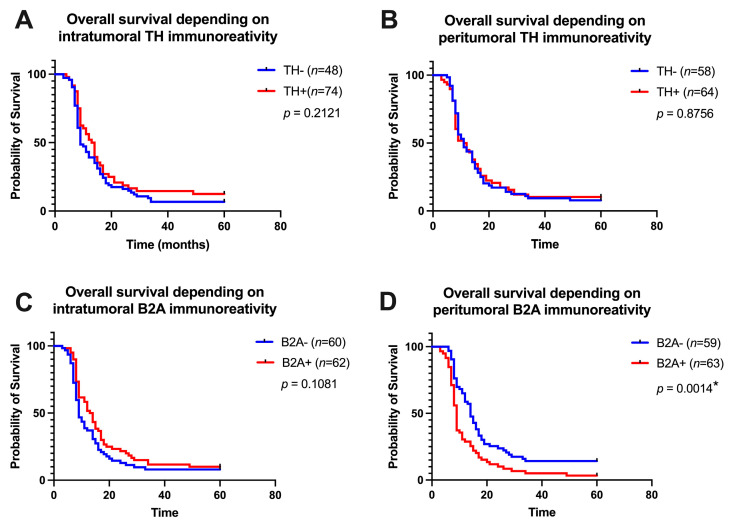
Overall survival of patients depending on tyrosine hydroxylase (TH) immunoreactivity intratumoral (**A**) and peritumoral (**B**). Additionally, overall survival of patients depending on beta 2 adrenoreceptors (B2A) immunoreactivity intratumoral (**C**) and peritumoral (**D**). P value with Log-rank test. * represent statistical significance.

To investigate the potential interaction between the immunoreactivity of tyrosine hydroxylase (TH), beta 2 adrenergic receptors (B2A), and clinicopathological outcomes, we used the median to classify each case as TH+, respectively, B2A+ (if it presented a value higher than the median). Firstly, the overall survival was analyzed according to TH and B2A immunoreactivity, both intratumorally and peritumoral (Figure 4). Only B2A immunoreactivity in the peritumoral pancreatic tissue influenced overall survival at 5 years of follow-up, thus B2A+ patients recorded a 5-year survival of only 3% compared to B2A− patients who recorded an overall survival at 5 years of follow-up up by 14% (HR = 1.758, 95% CI of ratio 1.297 to 2.938, *p* = 0.0004, Figure 4D). Thus, for the subsequent analysis, only the immunoreactivity of beta 2 adrenoreceptors was used, and each case was reclassified as B2A− if it registered a median of beta 2 adrenoreceptors in the peritumoral tissue lower than 750,000 in terms of IOD, or as B2A+ if it registered the median of beta 2 adrenoreceptors in peritumoral tissue greater than 750,000 in terms of IOD.

### 3.2. Baseline Clinicopathological Features

In Table 1, clinicopathological features are highlighted according to B2A immunoreactivity. Thus, we observed an association between the increased immunoreactivity of beta 2 adrenergic receptors and the age of patients younger than 60 years (37 patients—62.71%, Odds ratio 3.134, 95% CI 1.475 to 6.606, *p* = 0.0021), tumors moderately and respectively poorly differentiated (tumor grading G2 and G2 52 patients—54.17%, Odds ratio 3.208, 95% CI 1.235 to 8.034, *p* = 0.0157) but there was also a lack of response to first-line chemotherapy (46 patients—54.76%, Odds ratio 2.328, 95% CI 1.059 to 5.217, *p* = 0.0450).

### 3.3. Impact of Beta 2 Adrenoreceptors from Peritumoral Tissues in Pancreatic Cancer

**Figure 5:**
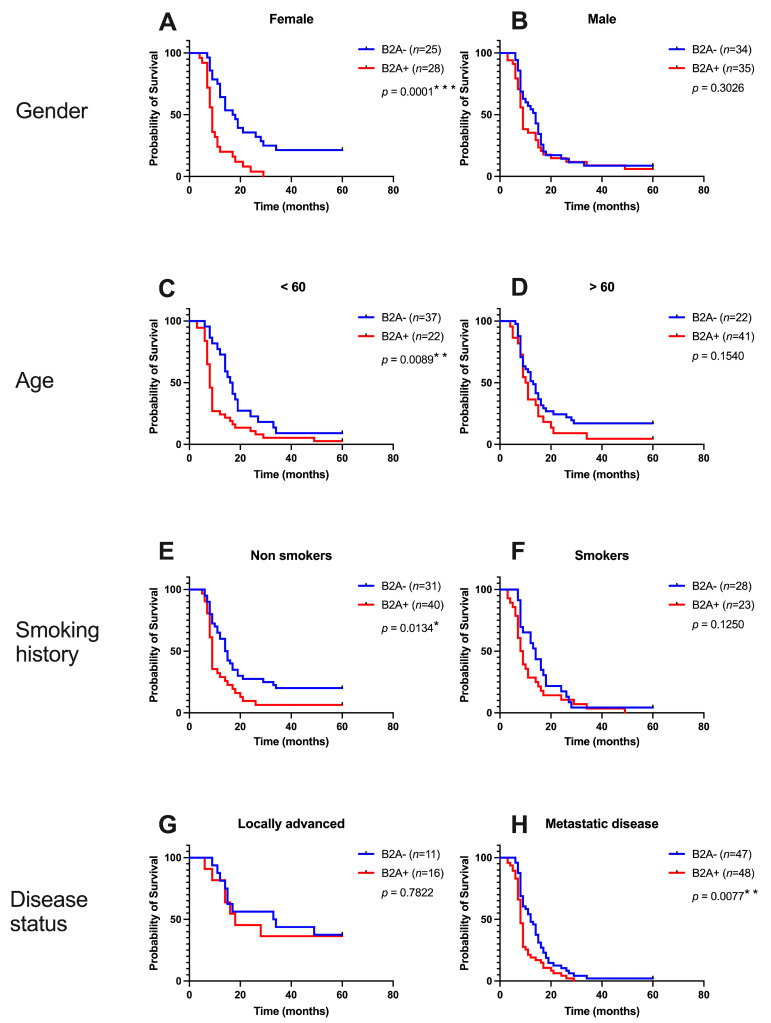
Kaplan–Meier overall survival curves depending on gender (**A**,**B**), age (**C**,**D**), smoking history (**E**,**F**), and disease status (**G**,**H**). *p* value with Log-rank test. * represents statistical significance, ** represents very significant and *** represents extremely significant.

The patients stratified according to the immunoreactivity of beta 2 adrenoreceptors in the peritumoral tissue were investigated using the Kaplan–Meier survival analysis, considering clinicopathological parameters. The median survival was 17.5 months in B2A− women compared to 9 months in B2A+ women (HR = 3.811, 95% CI 1.925 to 7.545, *p* = 0.0002), while in B2A− men, the median survival was 14 months in those with B2A− and only 9 months in those with B2A+. There was no statistical significance of overall survival in the case of men (HR = 1.318, 95% CI 0.7799 to 2.227, *p* = 0.3026). In terms of age, the young B2A− patients had a median survival of 16.5 months compared to the young B2A+ patients, where the median survival was only 8 months (HR = 2.125, 95% CI 1.208 to 3.739, *p* = 0.0089). The non-smoking patients B2A− (14.5 months vs. 9 months, HR= 2.050, 95% CI 1.161 to 3.620, *p* = 0.0134) as well as the patients with metastatic disease B2A− (12 months vs. 8 months) had a better survival: HR = 1.861, 95% CI 1.178 to 2.938, *p* = 0.0077). No statistically significant differences were recorded in terms of B2A immunoreactivity in patients with local disease and in patients who smoked. All these survival curves are shown in Figure 5.

**Figure 6:**
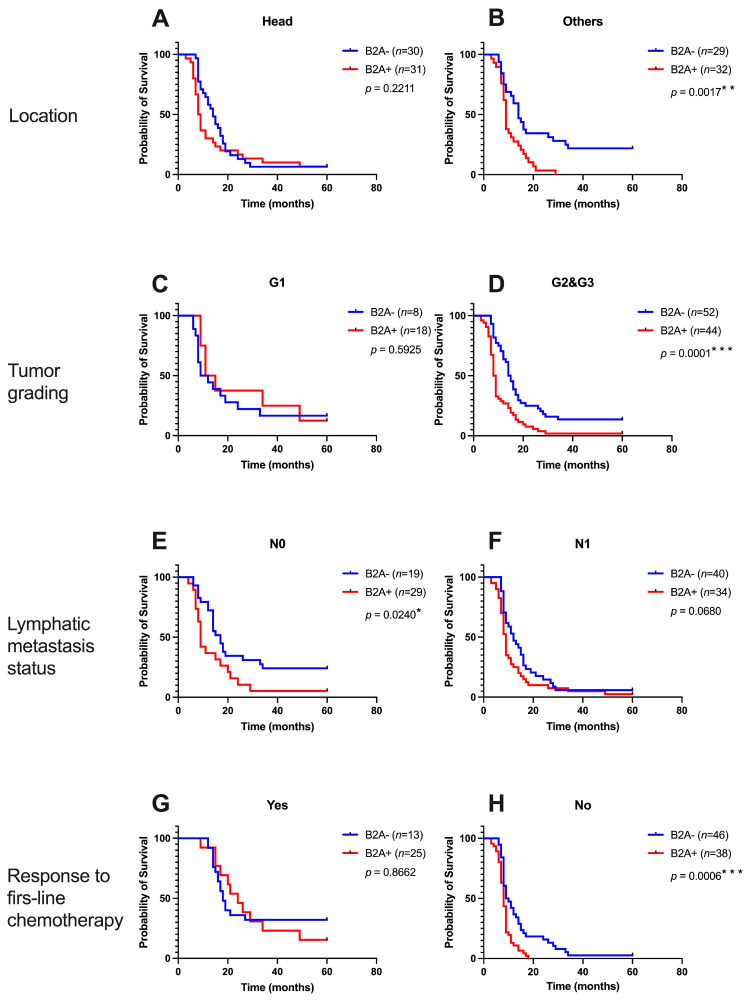
Kaplan–Meier overall survival curves depending on location (**A**,**B**), tumor grading (**C**,**D**), lymphatic metastasis status (**E**,**F**), and response to first-line chemotherapy (**G**,**H**). P value with Log-rank test. * represents statistical significance, ** represents very significant and *** represents extremely significant.

Figure 6 shows the Kaplan–Meier curves for overall survival depending on B2A immunoreactivity and tumor location, tumor grading, regional lymphatic metastases, and response to first-line chemotherapy. B2A− patients whose tumor was not located at the level of the head of the pancreas had a better survival time (14 months vs. 9 months, HR = 2.664, 95% CI 1.446 to 4.907, *p* = 0.0121), while patients with the tumor located at the level of the head of the pancreas did not register different survival curves depending on the immunoreactivity of B2A receptors (*p* = 0.2211). In terms of tumor differentiation, there were no statistically significant differences according to B2A receptor immunoreactivity in well-differentiated tumors, compared to moderately and poorly differentiated tumors, where B2A− patients had a better survival time (14.5 months vs. 8.5 months, HR = 2.433, 95% CI 1.537 to 3.852, *p* = 0.0001). Additionally, in patients with regional lymphatic metastases, there were no differences in survival compared to patients without regional lymphatic metastases, where the median survival was 17 months in B2A− patients vs. 9 months in B2A+ patients (HR = 2.273, 95% CI 1.114 to 4.638, *p* = 0.0240). The response to first-line chemotherapy did not influence survival according to B2A immunoreactivity, compared to the absence of response to first-line chemotherapy, although the median survival was only 9.5 months in B2A− vs. 8 months in B2A+ patients. Overall survival was better in B2A− patients (HR = 2.441, 95% CI 1.462 to 4.077, *p* = 0.0006).

## 4. Discussion

This study analyzed new perspectives of sympathetic neuronal alterations in pancreatic cancer. Firstly, a decrease in both the intratumoral sympathetic nerve fibers and the density of beta 2 adrenoreceptors was observed, which was proportional to the decrease in the degree of tumor differentiation. Moreover, an increase of beta 2 adrenoreceptors was observed in the peritumoral tissue, while the density of sympathetic fibers did not register a decrease or an increase, depending on tumor grading. Furthermore, the density of peritumoral beta 2 adrenoreceptors was correlated with reduced survival of patients with increased immunoreactivity for these receptors in the peritumoral tissue.

### 4.1. Alterations of Sympathetic Nerve Fibers

Our study revealed a significant decrease in pancreatic nerve fiber density in pancreatic cancer compared to the tumor microenvironment. The results were similar to those described in 2021 by Ferdoushi et al. [3], who noted a reduction in the percentage of sympathetic nerve fibers in tumors compared to sympathetic fibers in peritumoral nerves. In our study, we did not calculate the nerve cross-sectional area, although we determined the percentage of sympathetic nerve fibers in each nerve (% of the area of a nerve). Using this method, we did not observe an influence on the overall survival at 5 years of follow-up for the analyzed patients, except in the case of beta 2 adrenoreceptors, which also represents the element of novelty in our study. These neuronal alterations highlighted in our study could not be correlated with clinical outcomes due to their inhomogeneity. On the other hand, the data existing in the literature are contradictory. Ceyhan et al. showed that pancreatic sympathetic innervation was significantly altered in pancreatic cancer as well as in chronic pancreatitis, along with parasympathetic innervation [4]. This alteration was also highlighted in the case of intrapancreatic glial cells, a phenomenon that the authors refer to as a “neural remodeling” that produces “pancreatic neuropathy” [4]. A similar reduction in the intrapancreatic neural density and the number of nerves was observed in pancreatic ductal adenocarcinoma by Iwasaki et al. [6]. Moreover, this study highlighted that low intrapancreatic neural density in patients with pancreatic ductal adenocarcinoma represents a negative prognostic factor [6]. There are also studies that have shown that in the case of pancreatic cancer patients, an increase in neural density or the number of nerves is recorded [6,15,16]. This information tells us that the innervation of pancreatic cancer is still not well known, and research must continue to improve our understanding. Additionally, there are differences in methodology regarding the studies in apparent opposition, as mentioned earlier. In general, it is accepted that intratumoral nerves have a lower density towards the center of the tumor, where a desmoplastic stroma predominates [6]. These differences are attributed to an interaction between nerves, pancreatic neoplastic cells, and stromal cells, such as cancer-associated macrophages and fibroblasts [17,18,19,20]. This intratumoral pancreatic neuronal alteration can also be explained by the invasion capacity of pancreatic neoplastic cells; the exact molecular mechanisms that explain this neoplastic intrapancreatic neuronal remodeling are unknown thus far [21]. Interestingly, a recent study indicates that sensory neuronal ablation in a genetic model of pancreatic ductal adenocarcinoma reduces cancer initiation and progression [22]. The involvement of the autonomic nervous system in tumorigenesis has attracted the attention of researchers in other types of cancer as well. Thus, Magnon et al. showed that surgical or chemical ablation of the hypogastric nerves was associated with a reduction in tumorigenesis in a mouse model of prostate cancer, proposing that postganglionic sympathetic neurons regulate tumor initiation, while postganglionic parasympathetic neurons play a major role in tumor progression. [23]. In another visceral cancer model, Zhao et al. highlighted that surgical vagotomy decreases tumor progression in gastric cancer, increasing the effectiveness of chemotherapy [24].

### 4.2. Immunoreactivity of Beta 2 Adrenoreceptors

From a molecular point of view, the influence of the sympathetic nervous system was also evaluated by analyzing the immunoreactivity of beta 2 adrenoreceptors in relation to clinical outcomes. Similarly to the analysis of sympathetic nerve fibers, intratumorally, we observed a decrease in the immunoreactivity of beta 2 adrenoreceptors, but without correlations of this decrease with clinical outcomes, especially in relation to survival. It is interesting that the intratumoral density of these receptors was also analyzed in other types of cancer in which a correlation of the immunoreactivity of these receptors with negative prognostic factors was observed, such as colon cancer [25], but sympathetic influences correlated with poor prognosis were also highlighted in hepatocarcinoma [26] or stomach cancer [27]. Increased peritumoral immunoreactivity of these receptors showed a correlation with poor prognostic factors, such as overall survival at 5 years. It is well known that beta 2 adrenoreceptors are members of the G protein-coupled receptor superfamily, receptors that mediate the action of endogenous catecholamines in a wide variety of target cells [28,29]. In short, the activation of these receptors stimulates cell proliferation by activating the cyclic adenosine monophosphate (cAMP)/protein kinase A (PKA) pathways [28]. Moreover, recent studies suggest that the activation of beta 2 adrenoreceptors stimulates the subsequent activation of Src and Ras tyrosine kinases via the mitogen-activated protein kinase (MAPK) [30,31]. Thus, the activation of these kinases determines the transcriptional activation of some genes involved in tumor proliferation and antiapoptotic signaling cascades [32]. If the intracellular pathways mediated by beta 2 adrenoreceptors are well known, the question that arises is where do the agonists of these receptors come from in patients with pancreatic cancer? Do these patients have a higher level of endogenous catecholamines during the evolution of the neoplastic disease, or could they have had high levels of catecholamines even before the onset of this disease? Further functional studies are needed to answer these questions.

### 4.3. Limitations of the Study

The main limitation of our study is that it is an observational, descriptive study which was carried out retrospectively and which included a relatively small number of patients. Additionally, these observations were not reproduced on an animal model of pancreatic cancer. From a molecular point of view, not all intracellular signaling pathways were analyzed; we only investigated the immunoreactivity of beta 2 adrenoreceptors in these tissue samples, and we did not stimulate or inhibit them on normal pancreatic cell lines or pancreatic neoplastic tissue.

## 5. Conclusions

Sympathetic nerve fibers undergo neuronal remodeling in pancreatic cancer. Intratumorally, this is also accompanied by a decrease in immunoreactivity for beta 2 adrenoreceptors, but lacks exact correlations with clinical outcomes. However, the increased immunoreactivity of beta 2 adrenoreceptors in pancreatic peritumoral tissue represents a poor prognostic factor in pancreatic cancer. This observation highlights the role of the tumor microenvironment in the progression of pancreatic cancer and may constitute a future therapeutic target.

## Figures and Tables

**Table 1:** Baseline clinicopathological features depending on beta 2 adrenoreceptors (B2A) immunoreactivity.

		B2A(−)	B2A(+)	
Variable	Category	Value	Value	*p*
Age (years)	<60 (*n* = 59)	22 (37.29%)	37 (62.71%)	0.0021 *
	>60 (*n* = 63)	41 (65.08%)	22 (34.92%)	
Gender	Female (*n* = 53)	28 (52.83%)	25 (47.17%)	0.8564
	Male (*n* = 69)	35 (50.72%)	34 (49.28%)	
Smoking history	No (*n* = 71)	40 (56.34%)	31 (43.66%)	0.2713
	Yes (*n* = 51)	23 (45.10%)	28 (54.90%)	
Disease status	Locally advanced (*n* = 27)	16 (59.26%)	11 (40.74%)	0.5142
	Metastatic disease (*n* = 95)	48 (50.53%)	47 (49.47%)	
Location	Head (*n* = 61)	32 (52.46%)	29 (47.54%)	0.9999
	Others (*n* = 61)	31 (50.82%)	30 (49.18%)	
Tumor grading	G1 (*n* = 26)	19 (73.08%)	7 (26.92%)	0.0157 *
	G2&G3 (n= 96)	44 (45.83%)	52 (54.17%)	
Lymphatic metastasis status	N0 (*n* = 48)	29 (60.42%)	19 (39.58%)	0.1398
	N1 (*n* = 74)	34 (45.95%)	40 (54.05%%)	
Response to first-line chemotherapy	Yes (*n* = 38)	25 (65.79%)	13 (34.21%)	0.0450 *
	No (*n* = 84)	38 (45.24%)	46 (54.76%)	

* *p* < 0.05 on Fisher’s exact test.

## Data Availability

The data presented in this study are available on request from the corresponding authors.

## Supplementary Materials

The following supporting information can be downloaded at: https://www.mdpi.com/article/10.3390/cancers15113040/s1, Figure S1: Representative picture with sympathetic fibers in pancreatic peritumoral nerves immunoreactivity for tyrosine hydroxylase, 4×. Figure S2: Representative picturewith sympathetic fibers in pancreatic peritumoral nerves immunoreactivity for tyrosine hydroxylase, 10×. Figure S3: Representative picturewith sympathetic fibers in pancreatic intratumoral nerves immunoreactivity for tyrosine hydroxylase, 4×. Figure S4: Representative picture with sympathetic fibers in pancreatic intratumoral nerves immunoreactivity for tyrosine hydroxylase, 10×. Figure S5: Representative picture with beta 2 adrenoreceptors (brown color) in pancreatic tumor tissue (the black arrow) and in peritumoral tissue (the white arrow), 4×. Figure S5: Representative picture with beta 2 adrenoreceptors (brown color) in pancreatic tumor tissue (the black arrow) and in peritumoral tissue (the white arrow), 10×.
