# Supplementary material for: Clinical Outcomes Depending on Sympathetic Innervation in Pancreatic Cancer
Source: Cancers (Basel). 2023 Jun 2;15(11):3040. doi: 10.3390/cancers15113040 (PMC10252059; doi:10.3390/cancers15113040)
Supplement: Supplementary file 1 [file cancers-15-03040-s001.zip › cancers-2380730-supplementary.pdf]

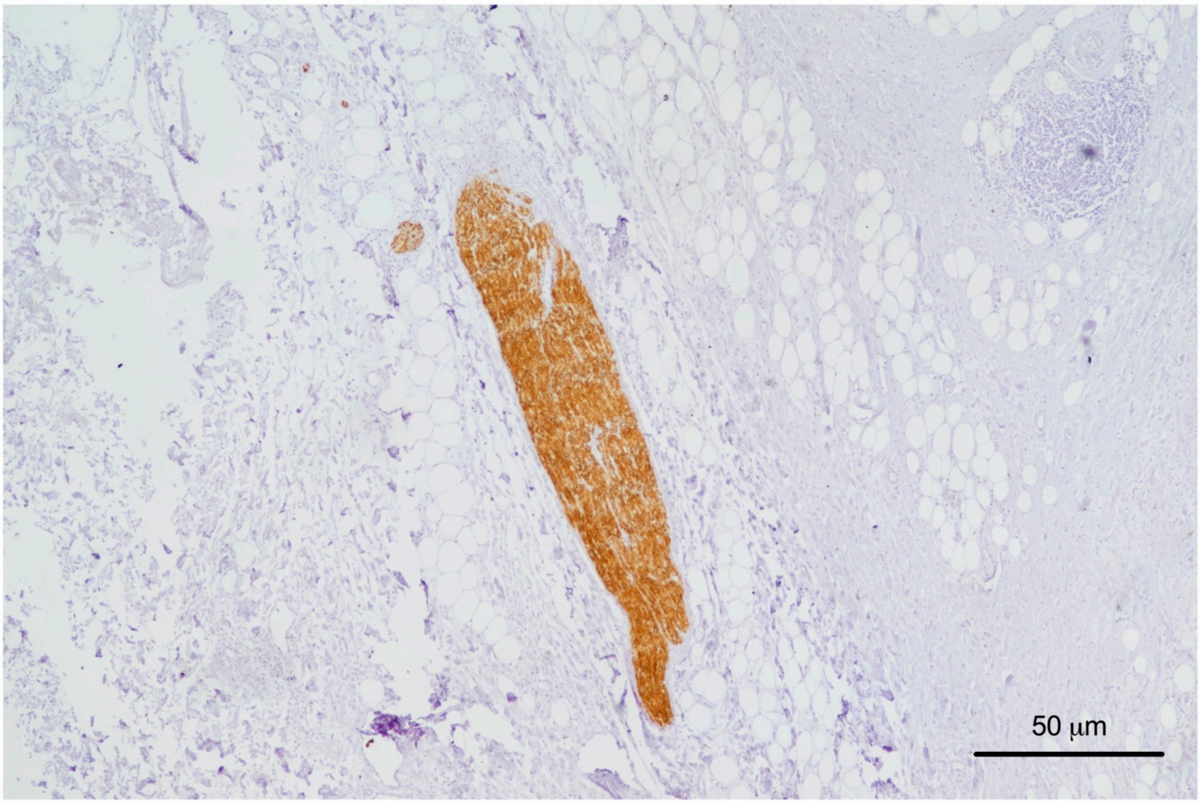

Figure S1. Representative picture with sympathetic fibers in pancreatic peritumoral nerves immunoreactivity for tyrosine hydroxylase, 4x.

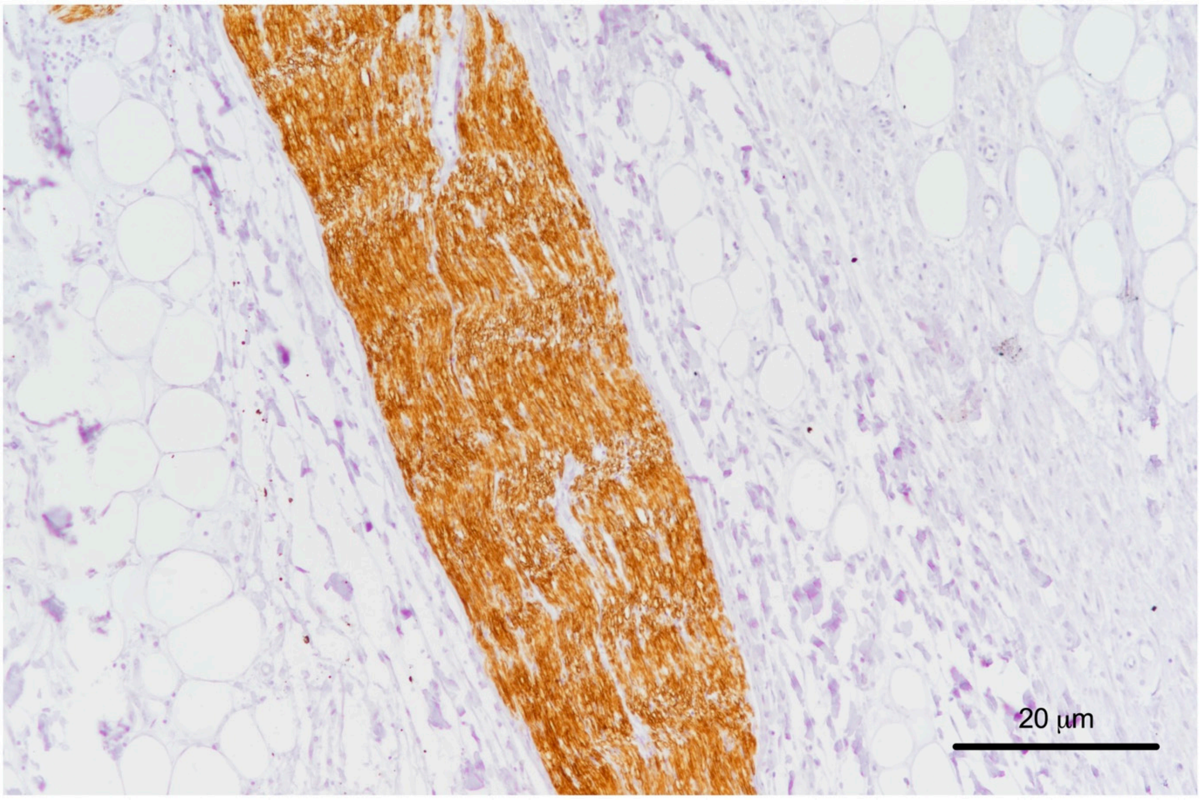

Figure S2. Representative picturewith sympathetic fibers in pancreatic peritumoral nerves immunoreactivity for tyrosine hydroxylase, 10x.

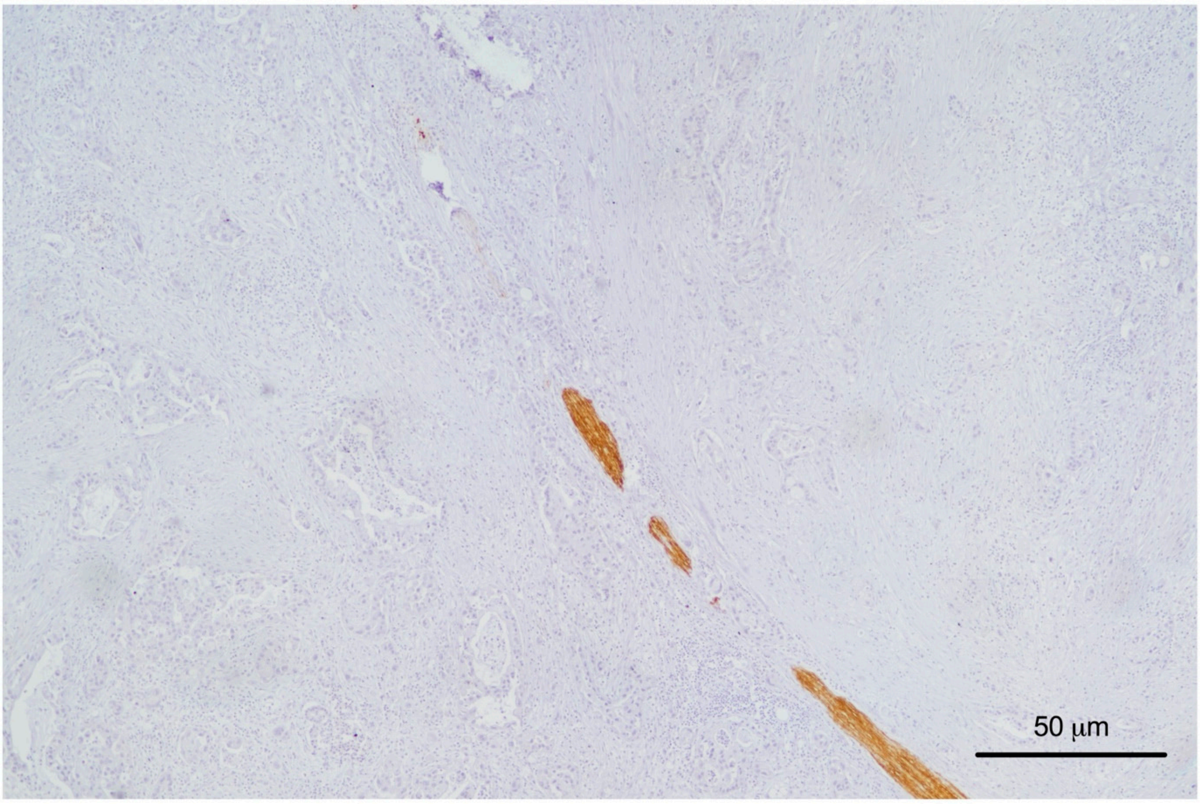

Figure S3. Representative picturewith sympathetic fibers in pancreatic intratumoral nerves immunoreactivity for tyrosine hydroxylase, 4x.

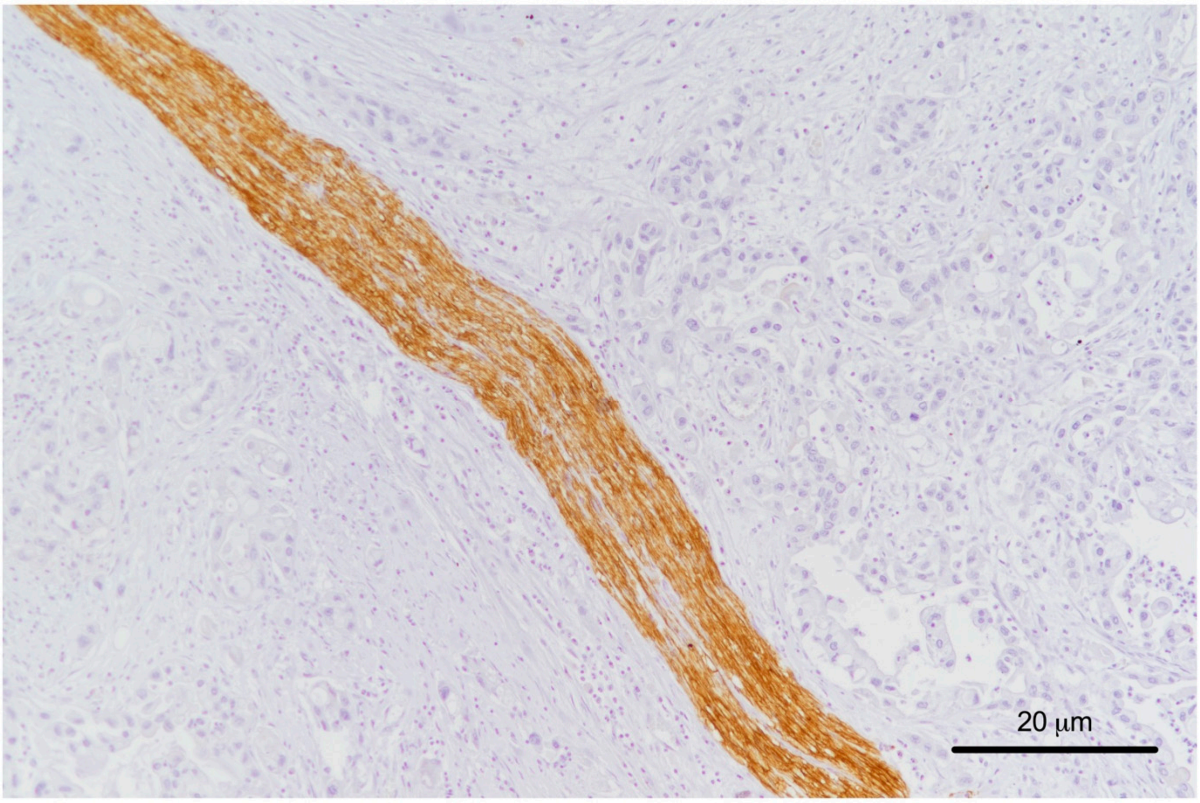

Figure S4. Representative picture with sympathetic fibers in pancreatic intratumoral nerves immunoreactivity for tyrosine hydroxylase, 10x.

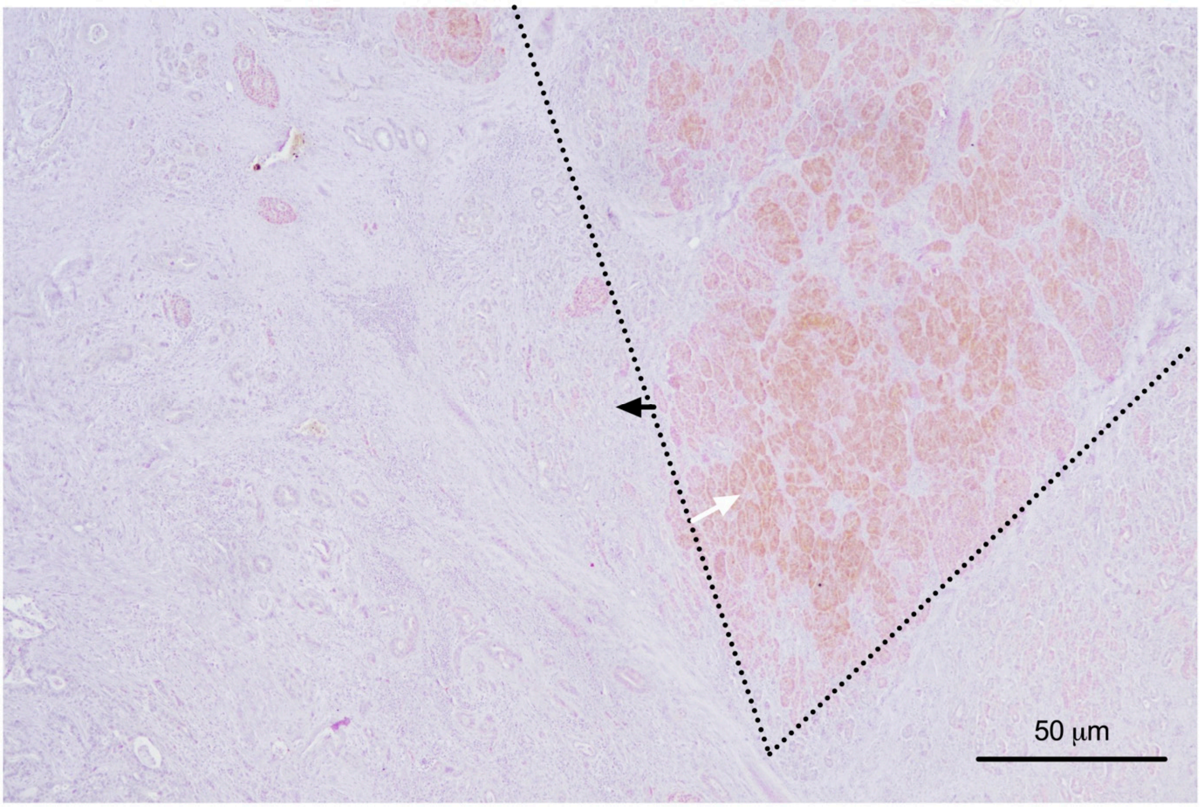

Figure S5. Representative picture with beta 2 adrenoreceptors (brown color) in pancreatic tumor tissue (the black arrow) and in peritumoral tissue (the white arrow), 4x.

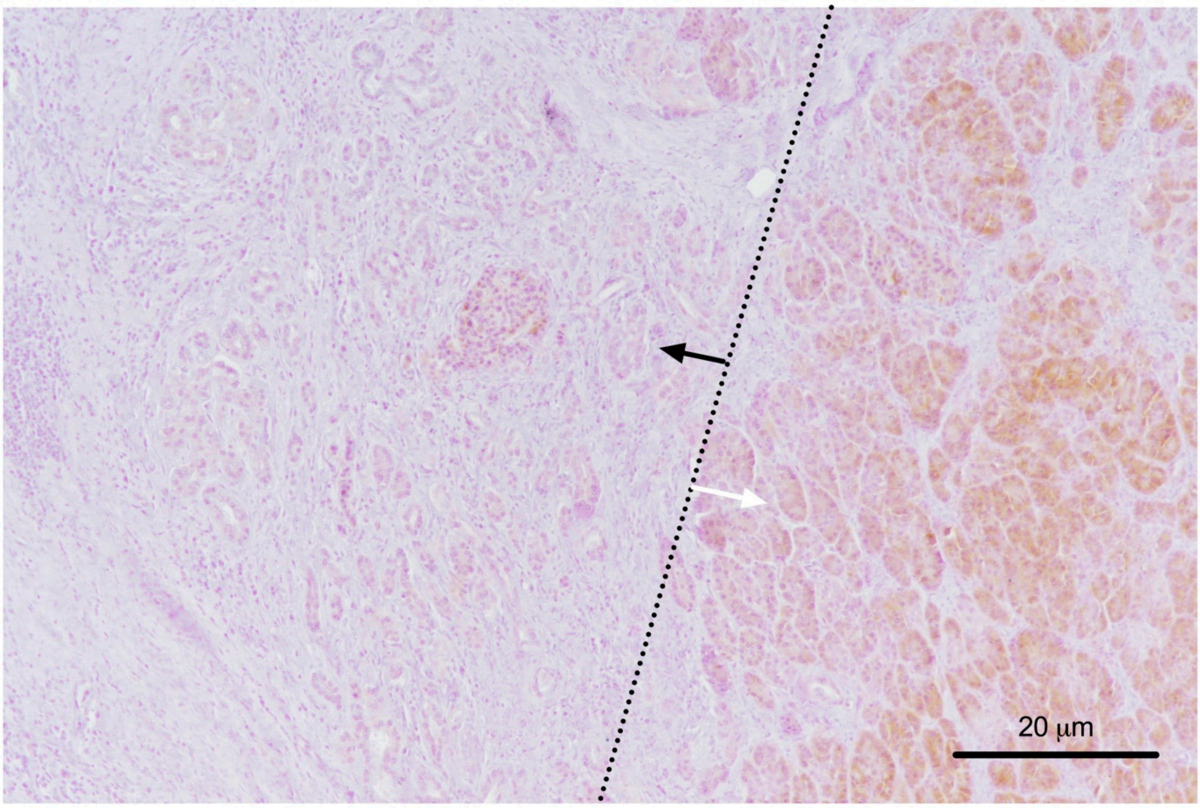

Figure S6. Representative picture with beta 2 adrenoreceptors (brown color) in pancreatic tumor tissue (the black arrow) and in peritumoral tissue (the white arrow), 10x.
